# Comparison of early and fully expanded blastocysts on pregnancy and birth outcomes in patients with fresh IVF/ICSI cycles: A retrospective cohort study

**DOI:** 10.1371/journal.pone.0308130

**Published:** 2024-08-29

**Authors:** Xiaoqin Pan, Yuanping Zhou, Liwen Shen

**Affiliations:** Department of Center for Reproductive Medicine, Huzhou Maternity & Child Health Care Hospital, Huzhou, Zhejiang, People’s Republic of China; Utah State University, UNITED STATES OF AMERICA

## Abstract

**Objective:**

To investigate the effect of the early blastocyst on pregnancy and birth outcomes in patients in vitro fertilization/(early rescue) intracytoplasmic sperm injection-embryo transfer [IVF/(early rescue)ICSI-ET] cycles.

**Methods:**

In this retrospective cohort study, 289 patients with single-blastocyst transfer within IVF/(early rescue)ICSI-ET treatment cycle were included and divided into the early (n = 48, Gardner stage = 1 or 2) and the fully expanded blastocyst (n = 241, Gardner stage ≥ 3) groups. The differences in pregnancy and birth outcomes between the two groups were compared.

**Results:**

There was no significant differences between the two groups in baseline indicators, including demographic characteristics and clinical treatment (*P*> 0.05).The clinical outcomes indicators in the early and the fully expanded blastocyst groups were compared, including the number of transferable embryos on the third day (D3)5.0 (4.0, 6.8) vs. 6.0 (5.0, 8.0) (*P* = 0.001), the number of remaining embryos frozen per cycle 1.0 (0.3, 2.0) vs. 3.0 (2.0, 5.0) (*P*<0.001); the number of cycles of unfrozen embryos 13/48 (27.1%) vs. 12/241 (5.0%) (*P*<0.001); the pregnancy outcome including the clinical pregnancy rate (CPR) 20/48 (41.7%) vs. 129/241 (53.5%) (*P*>0.05); the live birth rate (LBR)15/48 (31.3%) vs.106/241 (44.0%) (*P*>0.05). There were no significant differences in birth outcomes, such as gestational week of labor, mode of delivery, neonatal birth weight, height, Apgar score, sex ratio, and birth defects between the two groups (*P*>0.05).Multivariate binary logistic regression showed the same result, i.e., early blastocyst transfer in fresh cycle was not a risk factor for clinical pregnancy (OR = 0.516, 95% CI = 0.260–1.022) and live birth (OR = 0.521, 95% CI = 0.252–1.079).

**Conclusion:**

Compared with the fully expanded blastocyst group, the CPR and LBR in the early blastocyst group of the fresh transfer cycles were relatively ideal, and there were no significant differences in birth outcomes and neonatal status between the two groups.

## Introduction

The international definition of successful in vitro fertilization/(early rescue) intracytoplasmic sperm injection[IVF/(early rescue)ICSI] treatment is the absence of ovarian hyper stimulation syndrome (OHSS), singleton pregnancy, and healthy full-term live birth. Selection of a single blastocyst transfer with the most developmental potential is an important step, which increases the clinical pregnancy rate (CPR) and live birth rate (LBR) [1,2], and reduces the occurrence of OHSS [3], multiple pregnancies [4], and ectopic pregnancy [5].

At present, the simplest, effective and non-invasive method of evaluating embryos is morphology. Three parameters have been used in Gardner scoring system, including blastocoele expansion and hatch degree (EH), inner cell mass (ICM), and trophectoderm cells (TE). Each parameter correlates with the clinical outcome of fresh transfer cycles of blastocytes [6]. Embryos with higher scores are considered a priority in blastocyst transfer cycles.

However, even under the same conditions, embryos in extended culture can display heterogeneity in development rate [7], embryos from some patients do not reach the fully expanded blastocyst stage on day5 (D5) but only develop to the early blastocyst stage. The degree of blastocyst expansion is an independent factor in predictor of live birth rate [6], which is positively correlated with euploidy rate [8]. These factors limited the large-scale clinical implementation of D5 early blastocysts fresh transfer, therefore, resulting in a paucity of clinical studies on clinical pregnancy outcomes and a lack of knowledge about neonatal birth outcomes after D5 early blastocyst transfer.

In the present study, we have compared the effect of D5early blastocyst and fully expanded blastocysts groups on clinical pregnancy outcomes and neonatal birth outcomes in fresh cycles, resulting in no significant differences between two groups.

## Materials and methods

### Study design

This retrospective study analyzed the patients who received IVF/ICSI treatment from January 2019 to December 2021 at Reproductive Center of Huzhou Maternal and Child Health Care Hospital, Huzhou, Zhejiang, China. The study was approved by the Ethics Committee of Huzhou Maternal and Child Health Care Hospital (the approval number: 2023-J-064 on June 5, 2023). A total of 289 patients were enrolled in this study, including 48 cases in the early blastocysts group (Gardner stage = 1 or 2) and 241cases in the fully expanded blastocysts group(Gardner stage≥3). Inclusion Criteria: (1) age≤ 42 years; (2) single blastocysts was transferred within 5 days of fresh cycle. Exclusion Criteria: (1) uterine malformation; (2) severe intrauterine adhesions; (3) untreated hydrosalpinx; (4) pre-transfer genetic diagnosis/screening cycle; (5) patients with recurrent implantation failure (RIF) (defined as patients who have failed to conceive with 3 previous transfers of high-quality embryos) [9]; (6) multiple pregnancies; (7) cervical insufficiency. All the data accessed in June 2023. Blastocysts were scored according to the Gardner scoring system [8].

### Ovarian stimulation protocols

Individualized ovarian stimulation was performed by one of the following protocols: (1) gonadotropin-releasing hormone agonist (GnRH-a) long protocol, including a long-acting GnRH-a regimen in the early follicular phase and a long-acting regimen in the mid-luteal phase; (2) GnRH antagonist protocol. When two or more follicles with a diameter of ≥18 mm or at least three follicles with a diameter of ≥17 mm, the oocytes were eventually matured by intramuscular injection of 5,000 U or 10,000 U of human chorionic gonadotropin (HCG). After 34–36 hours, oocytes were collected under intravenous general anesthesia.

### Embryo culture and scoring

IVF or ICSI was performed 3–4 h after egg retrieved depending on patient’s indications. And the indication of early rescue ICSI was that patients with primary infertility were ≥3 years, and the infertility cause was non-tubal factor. Five to six hours (5–6 h) after insemination, the fertilized (i.e., the second polar body) eggs were observed. While the fertilization rate of eggs was less than 25%, the early rescue ICSI was immediately performed on the mature oocytes (MII) without the second polar body. Embryos were cultured superlatively in 25 μl droplets of G1 Plus medium (vitrolife) covered with mineral oil (ovoil, vitrolife) and incubated in an incubator at 37°C with a volume fraction of 6% CO_2_. Some embryos from day 3 were then cultured in G2 Plus medium (vitrolife) using a three-gas incubator (volume fraction 5% O_2_/6% CO_2_/89% N2) until day 5 or 6. On the first day (D1), fertilization was observed. From the 3^rd^ day (D3) after egg retrieval, cleaved embryos were graded according to 2011 Istanbul Consensus. The parameters included the number of blastomeres, morphology and cytoplasmic fragments, etc. Grade 1 embryos were uniform blastomere size and no cytoplasmic fragments; grade 2 uniform blastomere size, cytoplasmic fragments < 10%; grade 3 uneven blastomere size, cytoplasmic fragments < 20%; grade 4 embryos uniform or uneven blastomere size, cytoplasmic fragments 20%-50% [10]. On D3, cleaved embryos with 7–9 cells in grades 1 or 2 were considered to be of high quality. While the number of transferable cleaved embryos on D3 was < 4, 1 cleaved embryo was frozen and the rest were cultured to D5 or D6; Otherwise, all cleaved embryos were sequentially cultured and evaluated on D5 or D6 according to Gardner scoring system. Blastocysts were classified into stages 1to 6 according to the degree of blastocyst expansion and hatching (EH). Stage 1: a blastocyst with a blastocoel that is half of or greater than half of the volume of the embryo; Stage 2: a full blastocyst with a blastocoel completely filling the embryo; Stage 3: an expanded blastocyst with a blastocoel volume larger than that of the early embryo, with a thinning zona; Stage 4: a hatching blastocyst with the trophectoderm starting to herniate through the zona; Stage 5: a hatching blastocyst with the trophectoderm starting to herniate through the zona; Stage 6: a hatched blastocyst, in which the blastocyst has completely escaped from the zona.The development of ICM and TE was assessed as grade A, B, and C based on cell count and tightness. Grade A: tightly packed, many cells; Grade B: loosely grouped, several cells; Grade C: very few cells. For the purposes of this study, the early blastocyst refer to the degree of blastocyst expansion in stages 1 and 2. Transferable blastocyst: blastocyst expansion stage 3 or above, ICM and TE should have at least one item A or B. High-quality blastocyst: blastocyst expansion stage 3 or more, and ICM and TE both A or B. All blastocyst scores were performed by two embryologists.

### Fresh blastocyst transfer

On D5 after ovulation, one blastocyst was transferred according to the condition of the patient. From the date of egg collection, the corpus luteum was administered 2–3 mg of estradiol valerate (Bayer) and 20 mg of dydrogesterone (Abbott) orally twice daily, and 90 mg of Progesterone Sustained-release vaginal gel (Merck) once a day, until 8–10 weeks gestation.

### Observation parameter

Primary outcome, clinical pregnancy rate (CPR), was measured. Secondary outcomes included biochemical pregnancy, serum HCG level ≥ 10 IU/L 14 days after blastocysts transfer; clinical pregnancy, the presence of a gestational sac seen on transvaginal ultrasound 28 days after blastocysts transfer; premature delivery, delivery between 28 and 37 weeks of gestation; early miscarriage, the failure of pregnancy before 12 weeks; ectopic pregnancy, blastocysts implanted outside the uterine cavity; ongoing pregnancy, pregnancy lasting beyond 20 weeks; live birth rate (LBR), delivery at ≥28 weeks of gestation; gestational week of labor; delivery mode; low birth weight, newborn with birth weight < 2500 g; and birth defects, abnormalities in morphology, structure, function, metabolism, mental behavior, and other aspects during the development of blastocysts or fetuses, including congenital malformations, intellectual disabilities, and metabolic diseases.

### Statistical analysis

SPSS 25 software (IBM, New York, USA) was used for statistical analysis. The superlative data in this study were used for the Shapiro-wilk test, but the scholastic data did not conform to the normal distribution, so that the median and upper and lower quartiles [M(P25,P75)] were used for statistical analysis. The non-parametric Mann-Whitney test was used for statistical analysis. Count data were expressed as percentages (%), and statistical methods were using Chi-square test or Fisher’s exact probability method. The difference p <0.05 was statistically significant.Multivariate binary logistic regression was used to statistically analyze the correlation between early blastocyst transfer and pregnancy outcomes, and the odds ratio (OR) and 95% confidence index (CI) were calculated after adjusting for confounding factors.

## Results

### 1. Comparison of baseline data between the early and fully expanded blastocyst groups

The study included a total of 289 patients treated with IVF/(early rescue) ICSI, aged 22–42 years (mean = 29.9 years), 139 (48.1%) with primary infertility and 150 (51.9%) with secondary infertility, with infertility duration ranging from 1 to 15 years (mean = 3.3 years) and serum anti-mullerian hormone (AMH) levels ranging from 0.72 to 23.7 (mean = 5.74 ng/mL).The patients were divided into two groups, 48 cases in the early blastocyst group (13 cases in Gardner stage 1, 35 cases in stage 2) and 241 cases in the fully expanded blastocyst group(Gardner stage≥3) (186 cases of high-quality blastocysts and 55 cases of non-high-quality transferable blastocysts). There were no significant differences in maternal age, duration of infertility, type of infertility, serum AMH level, basal follicle-stimulating hormone (FSH), basal antral follicle count (AFC), and body mass index (BMI), treatment protocols, total Gn dose, serum estradiol (E2) level on the day of HCG injection, endometrial thickness on the day of HCG injection, number of eggs retrieved, and fertilization mode between the two groups (*P*>0.05) (Table 1). In the early and fully expanded blastocyst groups, the numbers of transferable embryos on D3 in the fresh cycle were 5.0 (4.0, 6.8) vs. 6.0 (5.0, 8.0) (*P* = 0.001), the remaining embryos cryopresed 1.0 (0.3, 2.0) vs. 3.0 (2.0, 5.0), (*P*<0.001), All 2 factors in the early blastocyst group were significantly lower than those in the fully expanded blastocyst group. and the cycles without frozen embryos were significantly higher than those in the fully expanded blastocyst group13 (27.1%) vs. 12 (5.0%) (*P*<0.001) (Table 1).

**Table 1:** Comparison of the demographic characteristics and clinical treatment of patients in the early blastocyst group and the fully expanded blastocyst group.

Variables	Early blastocyst group (n = 48)	Fully expanded blastocyst group (n = 241)	*P*-value
Age (years)	29.0 (26.3, 33.8)	29.0 (27.0, 32.0)	0.957
Infertility duration (years)	3.0 (2.0, 5.0)	3.0 (1.0, 4.5)	0.343
Infertility type (n, %)			0.357
Primary infertility	26 (54.2%)	113(46.9%)	
Secondary infertility	22 (45.8%)	128 (53.1%)	
Infertility cause (n, %)			0.212
Female factor	33 (68.8%)	195 (80.9%)	
Male factor	8 (16.7%)	21 (8.7%)	
Couple factor	4 (8.3%)	15 (6.2%)	
Unexplained factor	3 (6.3%)	10 (4.1%)	
AFC(n)	11.0 (8.0,18.0)	12.0 (9.0, 17.0)	0.469
AMH (ng/mL)	5.29 (2.91,6.65)	4.94 (3.47, 7.54)	0.297
bFSH (IU/L)	6.2 (5.3, 6.8)	6.0 (5.3,7.1)	0.916
BMI (kg/M^2^)	22.0 (19.6, 23.9)	22.7 (20.6, 25.1)	0.174
Ovarian stimulation protocol (n, %)			0.289
GnRH-a long protocol	40 (83.3%)	214 (88.8%)	
GnRH antagonist protocol	8 (16.7%)	27 (11.2%)	
Gonadotropin dosage(IU)	2100.0 (1650.0,2625.0)	2025.0 (1525.0,2700.0)	0.663
E2 on the day of HCG inj (pmol/L)	14809.0 (10192.0,23295.0)	14769.0 (10705.0,21848.0)	0.890
No. of oocytes retrieved (n)	11.5(9.0,15.8)	13.0 (10.0,16.0)	0.050
Fertilization methods (n, %)			0.055
IVF	35 (72.9%)	209 (86.7%)	
ICSI	11(22.9%)	27(11.2%)	
RESCUE	2 (4.2%)	5 (2.1%)	
No.of 2PN (n)	8.0 (6.0, 9.8)	9.0 (7.0, 12.0)	0.021
No. of transferable embryos on D3 (n)	5.0 (4.0, 6.8)	6.0 (5.0, 8.0)	0.001
No. of embryoscryo preserved (n)	1.0 (0.3, 2.0)	3.0 (2.0, 5.0)	0.001
No.of cycles without frozen embryos (n, %)	13 (27.1%)	12 (5.0%)	0.001
Endometrial thickness (mm)	10.5 (10.0,12.0)	10.0 (9.0,12.0)	0.188

Note: PN = Pronucleus.

### 2. Comparison of pregnancy outcomes between two groups

In the early and fully expanded blastocyst groups, pregnancy outcome indexes were shown as following: biochemical pregnancy rate (BCR) 22(45.8%) vs. 151(62.7%), (*P* = 0.030), clinical pregnancy rate (CPR) 20(41.7%) vs. 129(53.5%) (*P* = 0.133), early miscarriage rate (EMR) 4(20.0%) vs. 23(17.8%) (*P* = 0.999), ectopic pregnancy rate (EPR) 1(5.0%) vs. 0(0.0%) (*P* = 0.282), ongoing pregnancy rate (OPR) 15(31.3%) vs. 106(44.0%) (*P* = 0.102), live birth rate (LBR) 15(31.3%) vs. 106(44.0%) (*P* = 0.102) (Table 2). There were no significant differences in pregnancy outcome indexes except for the biochemical pregnancy rates between two groups.

**Table 2:** Comparison of pregnancy outcomes between the early blastocyst group and the fully expanded blastocyst group.

Variables	Early blastocysts Group (n = 48)	Fully expanded blastocysts group (n = 241)	*P*-value
Biochemical pregnancy n(%)	22 (45.8%)	151 (62.7%)	0.030
Clinical pregnancy n(%)	20 (41.7%)	129 (53.5%)	0.133
Ectopic pregnancy n(%)	1 (5.0%)	0 (0.0%)	0.282
Early miscarriage n(%)	4 (20.0%)	23 (17.8%)	0.999
Ongoing pregnancy n(%)	15 (31.3%)	106 (44.0%)	0.102
Live birth n(%)	15 (31.3%)	106 (44.0%)	0.102

Binary logistic regression analysis corrected for confounders (Age, infertility duration, infertility type, infertility cause, AFC, AMH, bFSH, BMI, Ovarian stimulation protocol, Gonadotropin dosage, E2 on the day of HCG inj, No of oocytes retrieved, fertilization methods, No of 2PN, No of transferable embryos on D3, No of cycles without frozen embryos), early blastocysts were a risk factor for biochemical pregnancy (OR = 0.390, 95%CI = 0.195–0.779, *P* = 0.008). However, it was not a risk factor for clinical pregnancy (OR = 0.516, 95%CI = 0.260–1.022), early pregnancy loss (OR = 1.185, 95%CI = 0.271–5.183), continuation of pregnancy (OR = 0.521, 95%CI = 0.0.252–1.079), and live birth (OR = 0.521, 95%CI = 0.252–1.079) (Table 3).

**Table 3:** Logistic regression analysis of the effect of early blastocyst transfer in fresh cycle on clinical pregnancy outcomes.

	B	Wald	OR (95%CI)	*P*
Biochemical pregnancy	-0.941	7.117	0.390 (0.195–0.779)	0.008
Clinical pregnancy	-0.662	3.604	0.516 (0.260–1.022)	0.058
Early miscarriage	0.170	0.051	1.185 (0.271–5.183)	0.821
Ongoing pregnancy	-0.651	3.080	0.521 (0.252–1.079)	0.079
Live birth	-0.651	3.080	0.521 (0.252–1.079)	0.079

### 3. Comparison of birth outcomes between two groups

The early and the Fully expanded blastocyst groups consisted of 15 (15/48, 31.3%) and 106 (106/241, 44.0%) live births, respectively. There were no significant differences in birth outcomes, including gestational week of labor, mode of delivery, neonatal birth weight, height, Apgar score, sex ratio, and birth defects, between the two groups (*P*>0.05) (Table 3). No birth defects were found in the early blastocyst group (0/15, 0.00%), and one case of congenital heart valve disease occurred in the fully expanded blastocyst group (1/106, 0.94%), without significant difference between the two groups (*P*>0.05) (Table 4).

**Table 4:** Comparison of birth outcomes between the two groups.

Variables	Early blastocysts group (n = 15)	Fully expanded blastocyst group (n = 106)	*P*-value
gestational week of labor (weeks)	39 (38,39)	39 (38,39)	0.419
Caesarean section n(%)	7 (46.7%)	56 (52.8%)	0.655
Premature delivery n(%)	0 (0.00%)	8 (7.5%)	0.585
Neonatal weight (g)	3150.0 (2750.0,3350.0)	3345.0 (2997.5,3582.5)	0.148
Low birth weight rate n(%)	0 (0.0%)	5 (4.7%)	0.868
Neonatal height (cm)	50.0 (48.0 50.0)	50.0 (48.0,50.0)	0.588
Apgar score (points)	10.0 (10.0, 10.0)	10.0 (10.0,10.0)	0.831
Male infant rate n (%)	6 (40.0%)	60 (6.6%)	0.227
Birth defect n(%)	0 (0.00%)	1 (0.94%)	0.999

## Discussion

Embryo transfer strategies have shifted from the cleavage embryo transfer to the single blastocyst transfer because of the technological improvement in vitro embryo culture system [11]. Although under similar conditions, the cultured blastocysts were with different success rates. A previous study has shown that about 30% of embryos develop slowly [12]. At present, the results of clinical treatment of D5 early blastocyst transfer with delayed development are still controversial. In some cases, the use of fresh cycle transplants, whenever possible, has given the potential impact that frozen embryo transfer (FET) cycles may have on the health of offspring [13]. On the other hand, early blastocyst transfer in a fresh cycle may affect the success rate of each transfer cycle. The recommendation is to continue culturing D5 early blastocyst to D6 or D7 fully expanded blastocyst for freezing, followed by transfer of blastocyst in subsequent FET cycles. However, there are no evidence-based studies to determine an advantageously therapeutic protocol. Our study focuses on early blastocyst transfer, which is performed only in patients who do not have transferable blastocysts (Gardner stage 3 or above). We have analyzed 289 patients received IVF/(early rescue) ICSI treatment, including 48 cases with the early blastocysts and 241 with the fully expanded blastocysts. By compared pregnancy outcomes between two groups, the clinical pregnancy rate (CPR) and live birth rate (LBR) in the early blastocyst group were slightly lower than those of the fully expanded blastocyst group but without significant difference. Multivariate binary logistic regression analysis shows the same result, i.e., early blastocyst transfer in the fresh cycle is not a risk factor for obtaining clinical pregnancy and live birth. CPR of single blastocyst transfer is similar to that of double cleavage embryo transfer. However, a meta-analysis has shown that CPR of cleavage embryo transfer is higher than that of blastocyst transfer [14]. The reason may be that some embryos with poor quality underestimated and abandoned during the culture of embryo. Our data about LBR are similar to the previous studies. Many studies have shown a positive correlation between morphological score of blastocyst and clinical pregnancy outcome, including that EH, ICM, and TE are associated with the outcome of single blastocyst transfer during the fresh cycle; the degree of blastocyst expansion is an independent factor for predicting LBR [15,16]. Although it is associated with relatively poorer clinical outcomes, the lower blastocyst expansion does not negate the clinical value of early blastocysts, which benefit at least a subset of patients in our study.

CPR and LBR of D6 and D7 blastocyst transfer compared with D5 blastocysts in FET cycles have reported significantly lower, concluding that D7 blastocyst transplantation is in the acceptable range although the pregnancy rate was lower [17]. The effect of embryo age on the clinical outcome of blastocyst transfer with both ICM and TE scores of grade C have been analyzed, concluding that D5 and D6 CC grade blastocysts had an acceptable pregnancy outcome [18]. The studies suggest that transferring the slower-developing or even lower-scoring blastocysts is a better option for patients who have difficulty obtaining high-quality blastocysts, which is consistent with the purpose of our study on early blastocyst transfer.

We have found that early blastocyst may benefit some patients. In this study, the number of D3 transferable embryos and remaining frozen embryos in the early blastocyst group was significantly less than that in the fully expanded blastocyst group. At the same time, the number of cycles without frozen embryos in the early blastocyst group was significantly higher than that in the fully expanded blastocyst group with statistically significant difference, suggesting that the early blastocyst group had fewer embryos available for the cycle, which could increase the utilization of embryos and reduce the risk of unavailable blastocysts being formed as a result of continued culture [19]. Therefore, the early blastocyst transfer might result in a more desirable pregnancy outcome.

Before transferring blastocysts in a FET cycle, which protocol is more suitable: transferring early blastocysts in a fresh cycle or continuing to culture blastocysts to the expansion stage? In two retrospective analyses of blastocysts with fresh cycle D5 slow development (Gardner stage 1and 2) and patients in the control group (Gardner stage ≥3) who continued to culture to D6 blastocysts that reached full expansion for resuscitation cycle transfer, there were no significant differences in clinical pregnancy rate or live birth rate between the two groups [20,21]. Therefore, transferring an early stage of the delayed blastocysts are recommended.

Combined with our study, we conclude that fresh transfer of early blastocysts is more benefit for patients because it shortens the treatment time period and avoids the health effects of the resuscitation cycle on the offspring [22–24], such as larger-than-fetal-age babies, adverse effects on early neurocognitive development of infants, significantly increased cancer risk in offspring in childhood, etc. Compared with Zhan’s study [21], the CPR and LBR of early balstocysts were slightly higher in our study, which may be due to the early blastocysts derived from cleaved embryos with different grades. Therefore, while we advocate that early blastocysts should be transferred in fresh cycles, we expect more high-quality studies in the future to investigate the factors affecting the success rate of early blastocyst in fresh transfer cycles, such as maternal age, embryonic dynamics, etc., with the aim of further screening the optimal range of applicability to improve the utilization and clinical outcomes of early blastocysts. However,the data of the low CPR and LBR of blastocyst with EH stage 1 in fresh transfer cycles at D5 suggest continued culture embryos to D6 or D7 [21]. Our study did not differentiate between early blastocysts with stage 1 and 2 due to the small sample size.

We also concern whether early blastocysts have an impact on neonatal outcomes. Some studies have shown that 35% of blastocysts meet freezing criteria with genetic abnormalities [22]. The degree of blastocyst expansion is significantly related to the euploidy rate. The blastocysts with delayed development have a high risk of aneuploidy [23,24]. Compared to blastocyst at stage 5 and 6, the euploidy rate of blastocysts at stage 1 and 2 was significantly decreased (81.1%vs.5.5%, *P* = 0.026) [25]. The aneuploid blastocyst is associated with embryo implantation failure and early pregnancy loss. In the present study, CPR in the earlyblastocyst group is lower than that in the fully expanded blastocyst group, which might be related to the aneuploid blastocyst. However, there is a lack of knowledge on the delayed blastocysts affecting birth outcomes. To our knowledge, our study is the first report to attempt to address this issue. In this study, there were 15 live births in the early blastocyst group and 106 live births in the fully expanded blastocyst group. There are no significant difference between the two groups in gestational age, delivery mode, birth weight, birth height, newborn sex ratio, and other variables. About the birth defects, there were no significant difference between the early blastocyst group (0 birth defects) and the fully expanded blastocyst group (1 birth defects diagnosed as congenital heart valve disease), which indicated that the high proportion aneuploidy in delayed blastocyst affected the treatment outcome mainly through natural elimination by embryo implantation failure or miscarriage. Therefore, while it is successfully implanted and continued until delivery, the delayed blastocyst does not have a significant effect on neonatal outcome and relatively safe.

This study can be used as a presentation of real-world clinical phenomena to help inform clinical decision-making. As a retrospective cohort analysis, the limitations include the small sample size, and the immediate and long-term risks that need a long follow-up period of the born offspring for the investigation.

## Conclusion

In conclusion, we have found that D5 early blastocyst transfer in fresh cycles can achieve better treatment outcomes, and there are no significant abnormalities in the birth outcomes and neonatal conditions for D5 early blastocyst transfer.

## Supporting information

S1 File Raw data. (XLSX)

S2 File Table 5. Comparison of pregnancy outcomes among the three different quality blastocyst groups.
(PDF)
