# Supplementary material for: Comparison of early and fully expanded blastocysts on pregnancy and birth outcomes in patients with fresh IVF/ICSI cycles: A retrospective cohort study
Source: PLoS One. 2024 Aug 29;19(8):e0308130. doi: 10.1371/journal.pone.0308130 (PMC11361670; doi:10.1371/journal.pone.0308130)
Supplement: S2 File — Comparison of pregnancy outcomes among the three different quality blastocyst groups. (PDF) [file pone.0308130.s002.pdf]

Table5.Comparison of pregnancy outcomes among the three different quality blastocyst groups

	Clinical pregnancy	Non- clinical pregnancy	X2	P	Live birth	Non- live birth	X2	P
Blastocyst type			3.370	0.185			2.670	0.263
Early blastocysts[n(%)]	20（41.7%）	28（58.3%）			15（31.3%）	33（68.8%）		
Low-quality blastocysts[n(%)]	26（47.3%）	29（52.7%）			24（43.6%）	31（56.4%）		
High-quality blastocysts[n(%)]	103（55.4%）	83（44.6%）			82（44.1%）	104（55.9%）		
